# Understanding the maternal and child health system response to payment for performance in Tanzania using a causal loop diagram approach

**DOI:** 10.1016/j.socscimed.2021.114277

**Published:** 2021-09

**Authors:** Rachel Cassidy, Andrada Tomoaia-Cotisel, Agnes Rwashana Semwanga, Peter Binyaruka, Zaid Chalabi, Karl Blanchet, Neha S. Singh, John Maiba, Josephine Borghi

**Affiliations:** a Department of Global Health and Development, London School of Hygiene and Tropical Medicine, 15-17, Tavistock Place, London, WC1H 9SH, UK; b RAND Corporation, Boston, MA, 02116, USA; c Information Systems Department, College of Computing and Information Sciences, Makerere University, P.O. Box 7062, Kampala, Uganda; d Ifakara Health Institute, PO Box 78373, Dar Es Salaam, Tanzania; e Department of Public Health, Environments and Society, London School of Hygiene and Tropical Medicine, 15-17, Tavistock Place, London, WC1H 9SH, UK; f Bartlett School of Environment, Energy and Resources, University College London, Central House, 14 Upper Woburn Place, London, WC1H 0NN, UK; g Geneva Centre of Humanitarian Studies, University of Geneva and the Graduate Institute, Rue Rothschild 22, 1211, Genève, Switzerland

**Keywords:** Health systems, Causal loop diagram, Payment for performance, Maternal and child health, Primary care, Evaluation, Tanzania

## Abstract

Payment for performance (P4P) has been employed in low and middle-income (LMIC) countries to improve quality and coverage of maternal and child health (MCH) services. However, there is a lack of consensus on how P4P affects health systems. There is a need to evaluate P4P effects on health systems using methods suitable for evaluating complex systems. We developed a causal loop diagram (CLD) to further understand the pathways to impact of P4P on delivery and uptake of MCH services in Tanzania. The CLD was developed and validated using qualitative data from a process evaluation of a P4P scheme in Tanzania, with additional stakeholder dialogue sought to strengthen confidence in the diagram. The CLD maps the interacting mechanisms involved in provider achievement of targets, reporting of health information, and population care seeking, and identifies those mechanisms affected by P4P. For example, the availability of drugs and medical commodities impacts not only provider achievement of P4P targets but also demand of services and is impacted by P4P through the availability of additional facility resources and the incentivisation of district managers to reduce drug stock outs. The CLD also identifies mechanisms key to facility achievement of targets but are not within the scope of the programme; the activities of health facility governing committees and community health workers, for example, are key to demand stimulation and effective resource use at the facility level but both groups were omitted from the incentive system. P4P design considerations generated from this work include appropriately incentivising the availability of drugs and staffing in facilities and those responsible for demand creation in communities. Further research using CLDs to study heath systems in LMIC is urgently needed to further our understanding of how systems respond to interventions and how to strengthen systems to deliver better coverage and quality of care.

## Highlights

• Holistic analysis key to avoiding suboptimal P4P performance.
• Availability of drugs is influenced by P4P and affects success of P4P.
• Need to incentivise groups outside facility that support service coverage.

## 1 Introduction

Payment for performance (P4P) programmes have been employed in many low- and middle-income countries (LMICs) to improve the quality and coverage of maternal and child health (MCH) services. Under P4P, health care providers, managers and/or organisations receive bonus payments that are tied to the delivery of pre-determined services or quality improvements (Mannion and Davies, 2008). The theoretical rationale for using financial incentives is to align incentives and behaviours of stakeholders within the health system in light of the agency relationships between managers, health care providers and patients, together with asymmetric information in these relationships (Fichera et al., 2014). Financial incentives are expected to motivate health workers to adhere to clinical care guidelines and increase the availability and quality of care delivered to patients (Gagné and Deci, 2005; Das et al., 2016). Many evaluations of P4P in LMIC have focused on estimating effects on elements within the health system, such as health worker job satisfaction (Shen et al., 2017; Engineer et al., 2016), health worker motivation (Shen et al., 2017; Engineer et al., 2016; Bhatnagar and George, 2016), availability of medical commodities (Das et al., 2016; Engineer et al., 2016; Bhatnagar and George, 2016), patient perceived quality of care (Das et al., 2016; Engineer et al., 2016; Paul et al., 2014) and accountability mechanisms (supervision of providers by managers (Bhatnagar and George, 2016; Paul et al., 2014; Mayumana et al., 2017) and community engagement in provision of services (Engineer et al., 2016; Mayumana et al., 2017)).

There has been less attention to the causal mechanisms through which P4P improves service delivery or coverage of health services. Causal mediation analysis was recently employed to unpack the mechanisms through which P4P improves service indicators in two low-income settings, isolating potential mediators of programme effect (Anselmi et al., 2017; Ngo et al., 2017). However, such analyses examine one-directional static single chains of causality, ignoring feedback mechanisms, overlooking dynamics in the health system as a whole, and disregard intrinsic time delays. We must consider the *holistic* impact of interventions on the health system, not just acknowledging that connections and mediators exist in isolation but how they affect each other over time. This knowledge is critical to understanding which design elements of P4P work and promote optimal health system behaviour (as intended) and which lead to suboptimal behaviour or negative unintended consequences, undermining programme success.

A recent realist review (Singh et al., 2021) identified pathways underpinning P4P effectiveness, including outreach activities to generate demand for services, greater availability of drugs and medical supplies and provider adherence to clinical guidelines. The review also pointed to relevant contextual factors underpinning programme effectiveness, including facility staffing levels and facility autonomy. Whilst informative, few of the studies included in this review were designed to evaluate pathways to P4P effectiveness or provide evidence of a link between a given mechanism and outcome.

Tools that derive from systems thinking methodologies can be used to better understand complex systems, such as health systems, and unpack the pathways to impact of interventions such as P4P (Borghi and Chalabi, 2017; Peters, 2014; Atun, 2012). Causal loop diagrams (CLDs) can identify and explore system problems and support decision making within health systems. They can also be used as a complementary tool to enhance other evaluation methods, such as realist evaluations, where there is a need to identify (and visualise) health system programme mechanisms and outcomes, and the context in which they are implemented (Singh et al., 2021; Renmans et al., 2020). CLDs are not a suitable choice for testing and modelling potential solutions to problems. Instead, system dynamics models, which often utilise CLDs in their development, are a better fit for this research need (de Savigny et al., 2017).

CLDs depict cause and effect relationships between variables in a system and provide a visual representation of system structure, capturing cyclic ‘looping’ feedback (Tomoaia-Cotisel et al., 2017). CLDs use arrows, where arrow polarity signifies the effect of changes in one variable on another. Delays in influence of one variable on another can be shown in CLDs using the symbol of two lines through an arrow. Reinforcing (R) and balancing (B) loops are identified in a CLD using numbered, circular arrows; reinforcing loops describe positive/amplified behaviour and balancing loops describe negative/stabilising behaviour. For more information on interpretation of CLDs, please see Appendix A. There has been a steady rise in the application of CLDs to evaluate the impact of policies on health systems in high income settings (Rashwan et al., 2015; Schoenenberger et al., 2016), most recently during the COVID-19 pandemic (Bradley et al., 2020; Sahin et al., 2020). To our knowledge, only four studies have used these methods to examine the effect of P4P interventions on health systems (Singh et al., 2021; Alonge et al., 2017; Meker and Barlas, 2015; Renmans et al., 2017), three in low-income settings (Singh et al., 2021; Alonge et al., 2017; Renmans et al., 2017).

The aim of this study was to develop a CLD to further understand the pathways to impact of P4P on delivery and uptake of MCH services in Tanzania, a low-income setting, and reflect on the insights gained from using this approach as compared to conventional evaluation methods. Tanzania was selected as a case study as it had implemented a P4P programme which was known to be effective in improving service uptake (Binyaruka et al., 2015), and resulted in health system improvements (provider kindness and greater drug availability) which mediated programme effects (Anselmi et al., 2017). There was also a wealth of evaluation data on the health system effects of the programme (Mayumana et al., 2017; Binyaruka et al., 2015, 2018a; Binyaruka and Borghi, 2017; Olafsdottir et al., 2014; Borghi et al., 2013; Binyaruka and Anselmi, 2020) to inform the CLD.

### 1.1 Study setting

Tanzania has experienced mixed progress in MCH over the last three decades (Afnan-Holmes et al., 2015) and implemented a P4P programme in 2011 as part of a concerted effort to make progress towards Millennium Development Goals 4 and 5 (Borghi et al., 2013). The design of the programme has been described extensively elsewhere (Binyaruka et al., 2015; Borghi et al., 2013), but a summary follows. The Ministry of Health and Social Welfare in Tanzania, with funding from the Norwegian Ministry of Foreign Affairs, introduced a P4P initiative in 2011 in the region of Pwani. To be eligible to participate in the programme, facilities had to provide MCH services, hold or open a bank account and provide facility performance data from the previous year (2010–2011), which was used to set initial MCH service coverage targets. Facilities were eligible for incentive payments if they met targets for each 6 month cycle; either a percentage increase on the previous cycle's performance or an absolute performance target (MoHSW Ministry of Health and Social Welfare, 2012; Binyaruka et al., 2018b) (see Appendix B). For primary health care facilities (dispensaries and health centres), 75 % of this payment was to be distributed among health workers at the facility and the remaining funds were to be spent on facility improvements/demand creation (25 %). Managers at the district and regional level who were responsible for supporting facilities and verifying facility performance data, the Council Health Management Team (CHMT) and Regional Health Management Team (RHMT), were also eligible for incentives (Appendix B).

## 2 Methods

### 2.1 Secondary data

We used qualitative data collected through a process evaluation during the Tanzania P4P programme (Borghi et al., 2013) to develop and validate a CLD (Table 1). These data describe how P4P was implemented in different facilities, factors that affected the success of the programme and potential unintended consequences (Borghi et al., 2013). Although secondary care facilities participated in the programme and consequent evaluation, due to programme design differences between providers, we focussed our evaluation on primary care facilities. Three rounds of data collection took place between December 2011–March 2013. Interviews were conducted in five of the seven districts in Pwani (Kibaha Town, Bagamoyo, Mkuranga, Kisarawe and Mafia island). Ten primary care health facilities were purposively sampled to reflect differences in level of care and ownership. Forty-three interviews were conducted with health workers, those in-charge of MCH care, those in-charge of facilities and members of the CHMT. Eight focus groups discussions (FGDs) were conducted with Health Facility Governing Committees (HFGC), CHMTs and health workers. Interviews were conducted in Swahili by four local social scientists working in pairs. All interviews were audio recorded and verbatim transcripts produced in Word, with transcripts translated to English.

**Table 1:** Description of secondary data used to develop and validate causal loop diagram, collected between December 2011–March 2013.

District	Facility/CHMT	Stakeholder Type	No. Of Interviews	No. Of FGDs
*District A*	Health Centre	Health worker	2	
	Dispensary	Health worker	3	
	CHMT	CHMT	5	1
*District B*	Health Centre	Health worker	4	
	Dispensary	Health worker	1	
		HFGC		2
	CHMT	CHMT	3	
*District C*	Dispensary	Health worker	1	
	Dispensary	Health worker	1	
		HFGC		1
	CHMT	CHMT	3	
*District D*	Health Centre	Health worker	2	
	Dispensary	Health worker	1	
	CHMT	CHMT	4	1
*District E*	Health Centre	Health worker	4	1
		HFGC		1
	Dispensary	Health worker	4	
	CHMT	CHMT	5	1
**Total**			**43**	**8**

Notes to Table: Council Health Management Team (CHMT) Focus group discussions (FGDs), Health Facility Governing Committee (HFGC).

### 2.2 Primary data

The CLD that was developed and validated using the secondary data described in the previous section was also validated by additional stakeholders in three rounds of data collection between March and December 2020. Twenty-one stakeholders who were closely involved with the evaluation and implementation of P4P in Tanzania were invited to interview via email communication. Interviews were conducted over Zoom due to COVID-19 travel restrictions. In the final round of data collection, stakeholders were also sent a flyer and link to a short film introducing the research and purpose of interviews. Stakeholders were asked to confirm the structure of the CLD or indicate if changes needed to be made to reflect their experience of P4P (see Appendix C for interview tool).

### 2.3 Creation of CLD

There were three steps to developing the CLD. First we used secondary data (Table 1) to develop individual CLDs (Kim and Andersen, 2012; Tomoaia-Cotisel, 2018) representing stakeholder understanding of how P4P affects their local health system. Second, individual CLDs were combined in a step-wise process (Tomoaia-Cotisel, 2018) resulting in a single CLD, an initial shared mental model of P4P's impact on the health system. Third, the combined CLD structure was validated to check to what extent additional stakeholders interviewed at the time of the original data collection agree on the structure of the system (Tomoaia-Cotisel, 2018) and to check to what extent additional stakeholders interviewed at the time of this study agreed that the CLD reflected their experience of the programme (Rwashana et al., 2014; Andersen et al., 2012).

### 2.4 Step 1: creation of individual CLDs

**Fig. 1:**
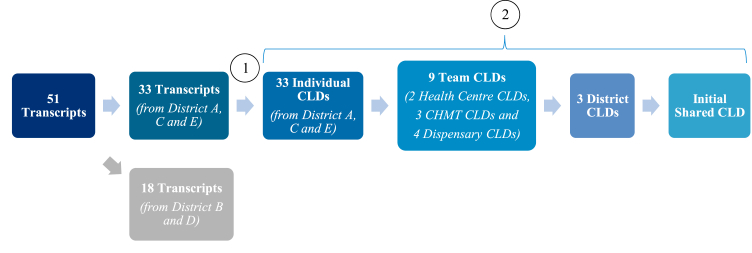
Process for creating (1) individual CLDs and (2) initial shared CLD. Notes to Figure: Step (1) Development of individual CLDs based on districts A, C and E (Table 1) and Step (2) Merging individual causal loop diagrams to create a single shared causal loop diagram. The 51 transcripts comprise of the transcripts from 43 individual interviews and from 8 focus group discussions. A CLD was developed for each transcript, one FGD transcript was used to develop one CLD. Adapted from (Tomoaia-Cotisel, 2018). Causal Loop Diagram (CLD), Council Health Management Team (CHMT).

**Fig. 2:**
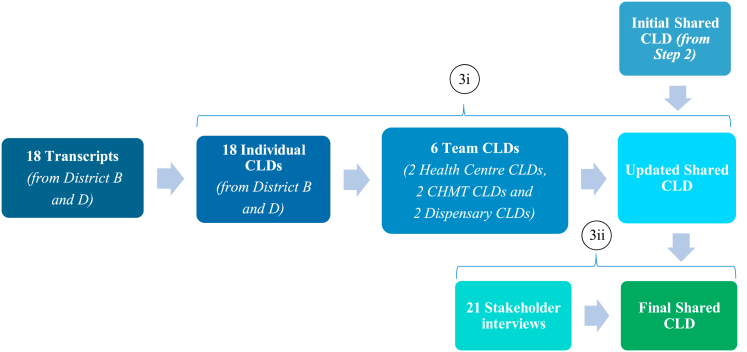
Validation of initial shared CLD. Notes to Figure: Step (3i) Comparison with team level CLDs that were not used to develop the shared CLD in the previous stage. Step (3ii) New stakeholder interviews to validate CLD structure. Adapted from (Tomoaia-Cotisel, 2018; Rwashana et al., 2014; Andersen et al., 2012). Causal Loop Diagram (CLD), Council Health Management Team (CHMT).

Interview and FGD transcripts were split into two groups; transcripts from districts A, C and E (Table 1) were used in Step 1 and 2 to develop an initial shared CLD (Fig. 1). These three districts (A, C and E) were selected to develop the initial shared CLD to represent variation in stakeholder group and geographical location. Transcripts from the remaining two districts (B and D) were used in Step 3 for initial validation of the CLD (Fig. 2).

To develop individual stakeholder-specific CLDs, cause and effect relationships from each transcript were elicited using Purposive Text Analysis (Kim and Andersen, 2012) adapted for CLDs (Tomoaia-Cotisel, 2018). Quotations were coded if they described events or scenarios that furthered understanding of how providers or health managers responded to the intervention in their facility or district, or demonstrated health system behaviour that facilitated or hindered facilities achieving P4P targets. Using this transformative process (Kim and Andersen, 2012; Tomoaia-Cotisel, 2018), coding was used to develop a single CLD for each stakeholder interview (using Excel to store this information and Vensim software (Ventana Systems Inc. Vens, 2015) to develop the CLD).

### 2.5 Step 2: creation of initial shared CLD

To create the initial shared CLD (Fig. 1), we combined individual CLDs into team CLDs (representative of facility or district management) through a process called CLD Combination (Tomoaia-Cotisel, 2018). Individual stakeholder CLDs within teams were ordered according to their level of ‘complexity’, in terms of the number of variables, links, loops and delays. The most complex CLD, the ‘anchor’ CLD, was compared to the second most complex CLD. The anchor CLD was altered to reflect new information in the second CLD, through a new segment of the CLD or refinement of existing content. This altered CLD was then compared to the third most complex CLD and so on until all individual stakeholder CLDs within that team had been combined into one team CLD. Where stakeholders described the same event but one CLD contained more information (a more complex loop), the complex loop was retained if the additional information was deemed necessary to understanding the behaviour of that particular part of the system. Next, we combined team CLDs into three district level CLDs using the same approach. Lastly, we combined the three-district level CLDs to create a shared (single) CLD.

### 2.6 Step 3: validation of initial shared CLD

Lastly, validation of the initial shared CLD was performed to ensure that critical input from each of the three stakeholder groups (health centres, dispensaries and CHMT) had not been lost or misinterpreted during the CLD development process (Fig. 2). Validation comprised of two stages: first, the initial shared CLD was validated to check to what extent additional teams interviewed at the time of the original data collection agree on the structure of the system (Tomoaia-Cotisel, 2018) and second, the updated shared CLD was validated to check to what extent additional stakeholders interviewed at the time of this study agreed that the CLD reflected their experience of the programme (Rwashana et al., 2014; Andersen et al., 2012).

In the first stage, we used interview and FGD data from districts B and D to generate 18 individual CLDs and then combined these individual CLDs into six team CLDs. We then compared each team level CLD to the initial shared CLD from Step 2, to see to what extent the team level CLDs confirmed the structure of the shared CLD or if any changes were required to the diagram (Tomoaia-Cotisel, 2018). Structural changes were made to the shared CLD to reflect the new elements identified in the team level CLDs (additional links and variables to expand concepts/loops already present in the CLD, modifications that increased understanding of supply of medical commodities at the facility).

In the second stage of validation, the CLD resulting from the first phase of validation was presented to twenty-one stakeholders closely involved with the evaluation and implementation of the P4P programme (Rwashana et al., 2014; Andersen et al., 2012). Stakeholders were asked if they recognised the structure and elements in the CLD and if any changes were needed to reflect their own experience of the health system and the P4P programme. This process aimed to minimise unconscious bias, to identify and amend any misinterpretation of data and elicit any further missing content. Structural changes were made to the CLD as a result of these interviews (strengthened understanding on use of facility and CHMT funding, additional drivers for health worker motivation, additional complexity included on pathways for addressing staffing levels at facilities).

### 2.7 Presentation of CLD

**Fig. 3:**
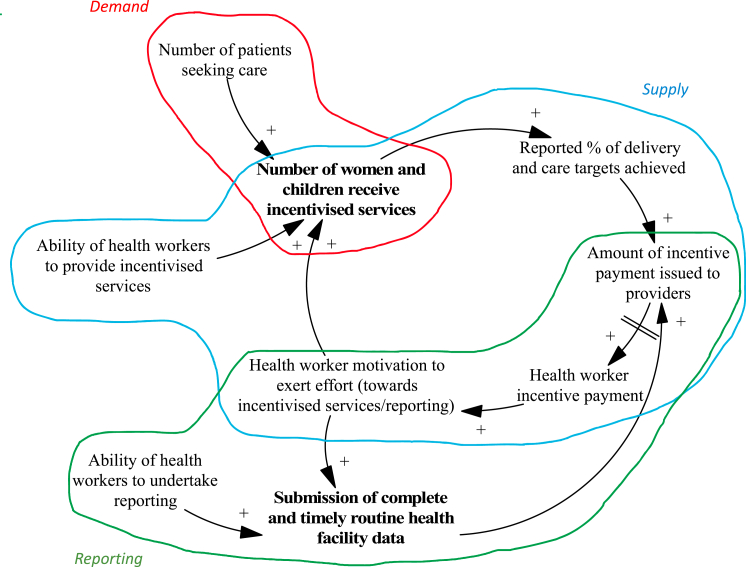
High level snapshot of causal loop diagram. Notes to Figure: Three main mechanisms responsible for provider achievement of (or failure to reach) targets during P4P are shown in different colours. Changes in the supply of services (blue), changes to facility reporting (green), and changes in demand for services (red).

We identify two categories of performance targets: ‘Number of women and children who receive incentivised services’ and ‘Submission of routine health facility data by providers’ (shown in **bold** in a high-level snapshot of CLD, Fig. 3). We identified three core mechanisms responsible for provider achievement of (or failure to reach) targets during the programme: (1) changes in the supply of services, (2) changes to facility reporting, and (3) changes in demand for services. We present an overview of each mechanism and the corresponding sections of the CLD (with the overall CLD shown in Appendix D), including stakeholder quotes from the qualitative data the CLD was developed from.

## 3 Results

### 3.1 Changes in the supply of services

**Fig. 4:**
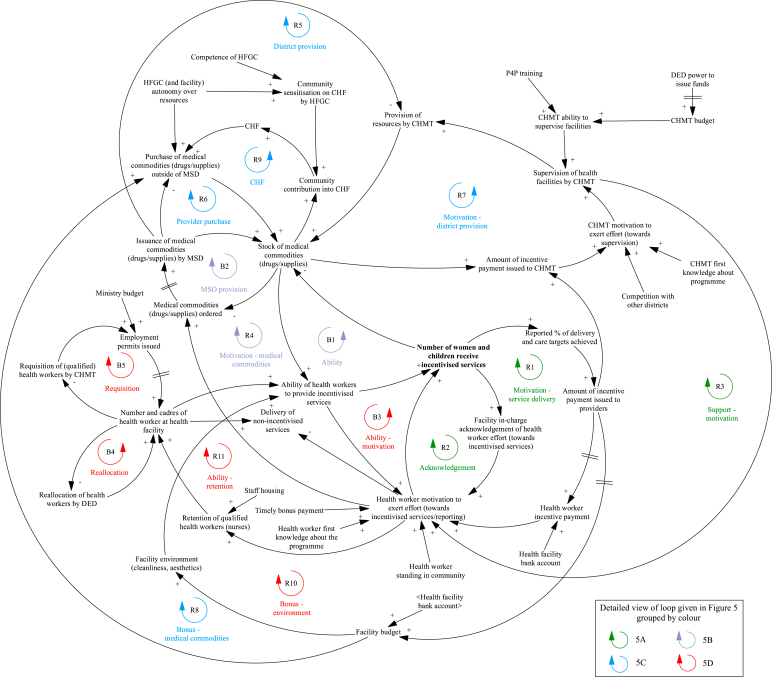
Changes in the supply of services during the programme that impact facility achievement of targets. Notes to Figure: Council Health Management Team (CHMT), Community Health Fund (CHF), District Executive Director (DED), Health Facility Governing Committee (HGFC), Medical Stores Department (MSD), Payment for performance (P4P).

**Fig. 5:**
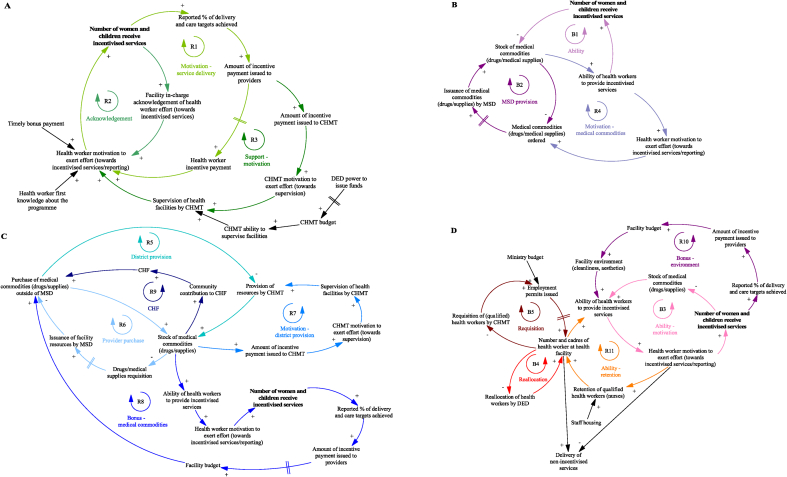
Detailed views of the mechanisms that result in changes in the supply of services during the programme and impact facility achievement of targets. Notes to Figure: Council Health Management Team (CHMT), Community Health Fund (CHF), District Executive Director (DED), Health Facility Governing Committee (HGFC), Medical Stores Department (MSD), Payment for performance (P4P).

The mechanisms that result in changes in the supply of services during the P4P programme are presented in Fig. 4, with individual loops shown in Fig. 5. The ‘motivation – service delivery’ loop (Fig. 5A, R1) is a virtuous cycle of growing action where incentive payments to providers increase health worker motivation to exert effort towards incentivised services. At the start of the programme before any payments are made, the promise of future bonus payments motivates health workers to achieve targets. On receiving the P4P incentive payment, health workers feel further motivated to reach targets. This initial boost and then sustained level of motivation is dependent on bonus payments being made on time; where payments to facilities are delayed (a common issue during the first year of the scheme) staff become frustrated and apathetic about the programme. Health workers also feel motivated to continually exert effort where their exertion is recognised by those in senior roles at the facility (Fig. 5A, R2) and where supervision visits by the CHMT are taking place (Fig. 5A, R3), as this makes health workers feel valued. However, the CHMT can only perform supportive supervision where funds for per diems and transport are available.

Health worker motivation to deliver incentivised services leads to timely requisition of medical commodities as seen in Fig. 5B, R4. As shown in the 'ability' loop (Fig. 5B, B1), providers can only deliver incentivised services where there are adequate levels of medical commodities. Providers become proactive in their requisition of these items in an effort to reduce stockouts; to ensure drugs and supplies are sent to the facility, requests need to be submitted within a strict timeframe. Requests are sent to the district pharmacist who liaises with the Medical Stores Department (MSD) (an autonomous government department) to procure drugs and medical supplies (Fig. 5B, B2). Delays and missing drugs in orders received from the MSD leads to failure of P4P service delivery targets if facilities are unable to procure from another source:

“One of the indicators was vaccines they are supposed to be given, but there are no vaccines to offer, at the end the facility will not score but that is not the facility's fault, it is somebody's fault. You may find (…) that the MSD does not supply all the drugs requested (….)”.

District level stakeholder, January 2012.

When the MSD are unable to fulfil an order, two virtuous cycles of growing action become dominant; CHMT provision of resources and facility purchase of drugs/medical supplies outside of MSD. The reduction of drug and medical supply stockouts at the facility level is an incentivised indicator for CHMT, through which CHMT members are motivated to support facilities (Fig. 5C, R7) and provide medicine and medical equipment where needed (Fig. 5C, R5). Facilities also use their own funds to purchase medicine and medical supplies from other sources (outside of the MSD) where needed (Fig. 5C, R6):

“There are changes, we used to get few drugs but since P4P started there is an improvement, if we get problems, we face our doctor (in charge) we use (…) P4P money to buy drugs. We take this opportunity to ask him to identify unavailable drugs in the facility then we buy them”.

**Fig. 6:**
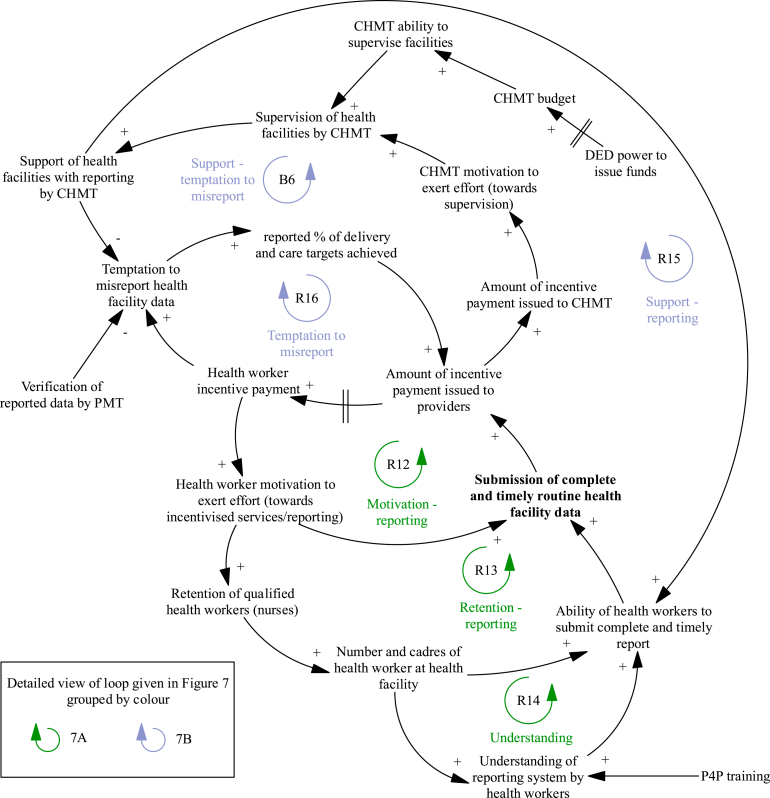
Changes to facility reporting during the programme that impact facility achievement of targets. Notes to Figure: Council Health Management Team (CHMT), District Executive Director (DED), Payment for performance (P4P), Pilot Management Team (PMT).

Facility level stakeholder, July 2012.

However, this is dependent on health providers having funding available (achieving P4P targets and receiving bonus payments) (Fig. 5C, R8), facilities setting up and having access to a bank account and an active HFGC. The HFGC, comprised of community members and health workers, support provider decision-making on use of funds at the facility and approve the release of funds. An additional source of funding outside of P4P that can be used by facilities to purchase medicine and medical supplies is the Community Health Fund (CHF) (Fig. 5C, R9). Providers saw this voluntary health insurance scheme as an opportunity to raise additional funds for service delivery (as premium revenue is kept by the facility) and increase the likelihood of achieving P4P targets (see ‘mechanisms that result in changes in the demand for services’ section for further details on the Fund).

Health worker motivation is tied to worker ability to deliver services. Where there are shortages in medicine, medical supplies (Fig. 5D, B3) and inadequacies in the facility environment (old mattresses, lack of cleaning equipment) (Fig. 5D, R10) impeding health worker ability to deliver services, health workers feel frustrated and demotivated, affecting staffing levels at facilities:

“They are frustrated by this (…) they had a medical doctor there, but he was not happy that he was sent to a facility that did not have a lot of equipment. He could not practice the skills he received during his training … so he was frustrated to the extent that he was planning to leave”.

Programme evaluation researcher, November 2020.

Adequate staffing levels and variety in the cadre of staff ensure an appropriate skill mix at the facility, to deliver more specialised services such as delivery care (Fig. 5D, R11). There were concerns that in facilities with depleted staffing levels, health worker motivation to deliver incentivised services and achieve reporting targets would result in task-shifting away from non-incentivised services (illustrated in Fig. 5D). To address vacancies the District Executive Director would reallocate staff to facilities in need (Fig. 5D, B4) and the CHMT request funding/permits for new staff (Fig. 5D, B5).

### 3.2 Changes to facility reporting

**Fig. 7:**
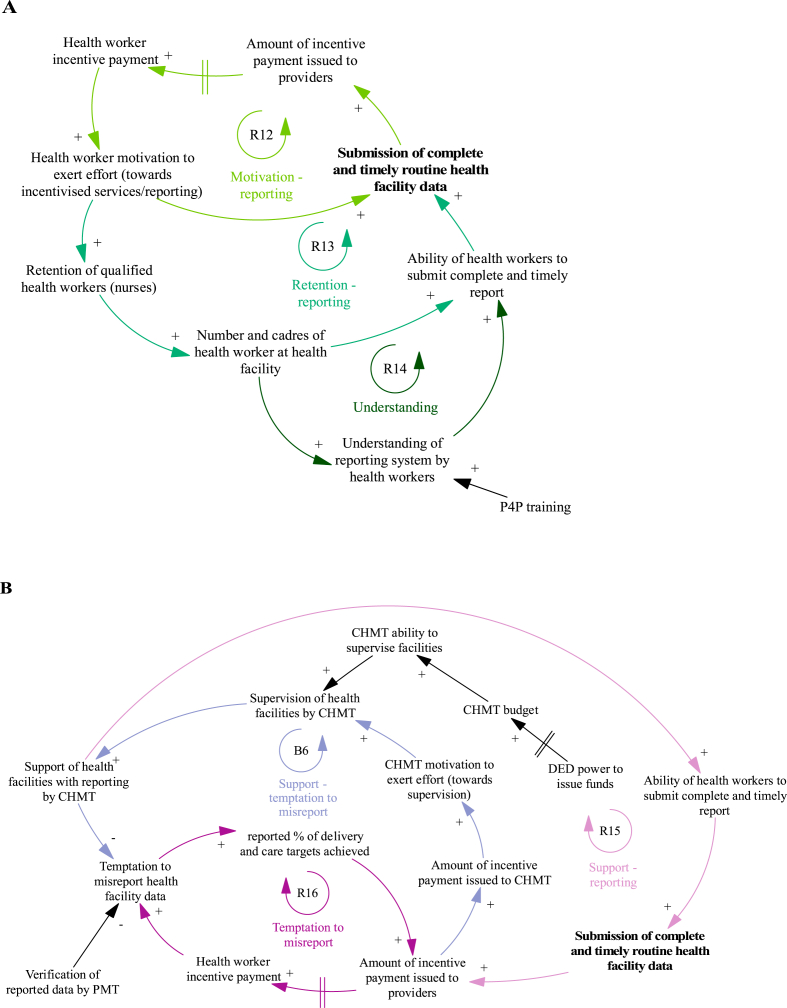
Detailed views of the mechanisms that result in changes to facility reporting during the programme and impact facility achievement of targets. Notes to Figure: Council Health Management Team (CHMT), District Executive Director (DED), Payment for performance (P4P), Pilot Management Team (PMT).

The mechanisms that result in changes to facility reporting during the P4P programme are presented in Fig. 6, with individual loops shown in Fig. 7. The ‘motivation – reporting’ loop (Fig. 7A, R12) is a virtuous cycle of growing action where incentive payments to providers increase health worker reporting of facility activity to the CHMT. This task can take considerable time, and facilities need adequate staffing to achieve this target alongside service delivery (Fig. 7A, R13) (with mechanisms for addressing staffing levels discussed in the previous section):

“Effort is done, we are expecting to get money in the third round, what was causing us not to get the money was the failure of submitting reports, the facility had one nurse. She said that she was overloaded, but since I arrived here the first thing I did was to make sure we submit reports”.

Facility level stakeholder, July 2012.

The ‘understanding’ loop (Fig. 7A, R14) is another virtuous cycle of growing action where health worker ability to undertake reporting is dependent on their knowledge of the reporting system. Health workers are sent for training at the start of the programme including on what routine health facility data should be reported to the CHMT. In facilities with high staff turnover, the training knowledge was lost with providers unable to achieve this target.

District manager (CHMT) incentives are partly driven by completeness of provider reporting (Fig. 7B, R15). CHMT members advise providers on record keeping and reporting during supervision visits. This offsets lack of provider knowledge (Fig. 7A, R14). In cases where facilities are unable to physically submit reports (due to lack of funds, transport or staff) the CHMT collect reports to support timely submission. The bonus payments encouraged district managers to make supervision visits:

“The bonus is like a carrot we have to run for it (…) we are trying to improve our systems as time goes on (…). So, we decided to start collecting report(s) because we discovered this will be very helpful to us. Though we face transport problem(s), I remember the last trip I went for supervision (I) was not paid, I spent my own money from my pocket because the budget for supervision was very minimal (…)”.

District level stakeholder, January 2012.

The ‘temptation to misreport’ loop (Fig. 7B, R16) is a vicious cycle illustrating the temptation to game the system and record higher levels of service delivery than actually provided to achieve higher incentive payments. Where mis-reporting is suspected during verification visits, an investigation and potential suspension of facility and CHMT incentive payments is implemented. CHMT supervision visits act as a deterrent for misreporting (Fig. 7B, B6); district managers compare reported data with facility records to ensure reported performance is accurate.

### 3.3 Changes in demand for services

**Fig. 8:**
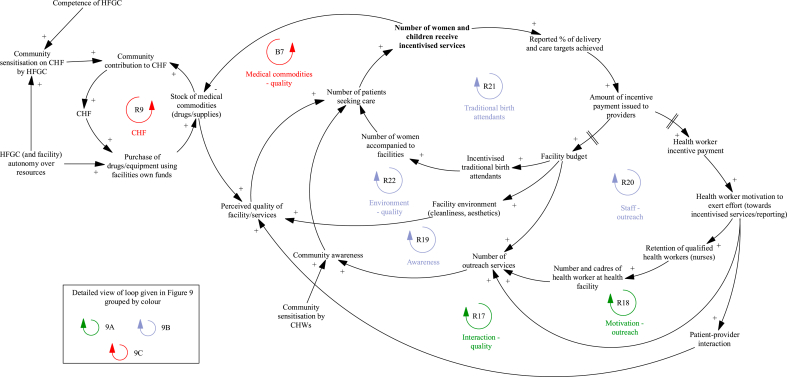
Changes in demand for services during the programme that impact facility achievement of targets. Notes to Figure: Council Health Management Team (CHMT), Community Health Fund (CHF), Community Health Workers (CHWs), District Executive Director (DED), Health Facility Governing Committee (HGFC).

**Fig. 9:**
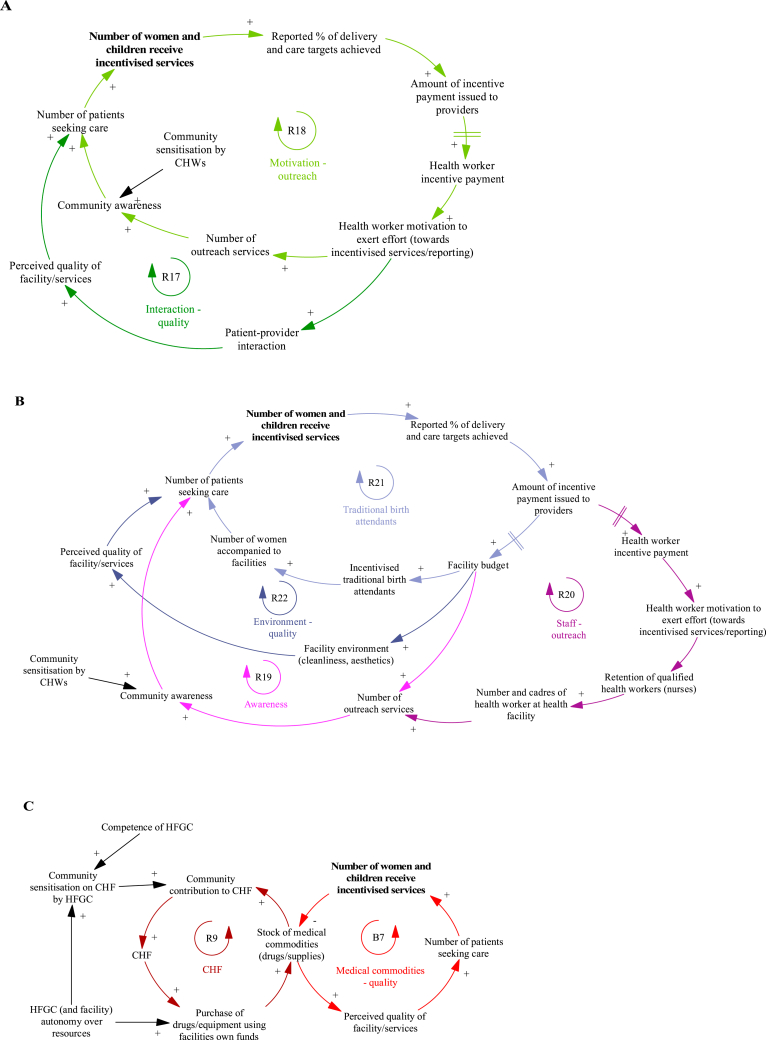
Detailed views of the mechanisms that result in changes in demand for services during the programme and impact facility achievement of targets. Notes to Figure: Council Health Management Team (CHMT), Community Health Fund (CHF), Community Health Workers (CHWs), District Executive Director (DED), Health Facility Governing Committee (HGFC).

The mechanisms that result in changes in demand for services during the P4P programme are presented in Fig. 8, with individual loops shown in Fig. 9. Improved patient-provider interaction (perceived kindness and respect from health workers) observed during P4P leads to an increase in the patient perceived quality of services (Fig. 9A, R17) and facility reputation, affecting the care seeking of other women.

“… workers are very polit(e) and kind to patients not like before. This surprise[s] the pregnant mothers, it is not like before when the workers were abusing them. Through P4P the pregnant mothers get good serve [health services] so she may tell her fellow [women] to come to the facility too [so] finally many of them will come to deliver [their babies] in the facility”.

Facility level stakeholder, December 2011.

A key mechanism to boost demand for services involves additional outreach activities carried out by providers in the community (Fig. 9A, R18). In some settings, this includes both community sensitisation activities and use of a mobile clinic to offer immunization services. The additional interaction between patients and providers in the community provides the opportunity to raise awareness of services offered at the facility and build trust between women in the community and facility workers, resulting in greater service uptake. Although health workers feel motivated to perform outreach services, their ability to do so is heavily dependent on funding for transport/fuel (Fig. 9B, R19) and adequate staffing levels (Fig. 9B, R20). The work of community health workers (CHWs) in engaging community members and promoting health education is critical to raising awareness of health issues and available services (Fig. 9A):

“You know the CHW, normally they come from the same community, and (…) are trusted people in that community. The community people are the one(s) who have chosen that person to be a community health worker (…) so if anything, if that CHW tells the community about maybe malaria, they trust it through the CHW more than anybody else (…) sensitisation becomes easier because it is their own people who tell the story”.

National level stakeholder, November 2020.

Other mechanisms for increasing demand include improving the facility environment (Fig. 9B, R22) and incentivising traditional birth attendants (Fig. 9B, R21). Providers used their bonus payments to purchase cleaning products, mattresses and other items to improve facility cleanliness and aesthetics. The facility environment is expected to impact patient perceived quality of services and decision to seek care, with improvements due to P4P, likely to increase demand from patients. Another innovative method employed by some facilities is to incentivise traditional birth attendants to boost the number of patients seeking care; incentive payments awarded to facilities who had increased their service performance are partially redistributed to traditional birth attendants who accompany women to attend facilities for institutional deliveries.

A key element that feeds into patient perceived quality of services and decision to seek care is availability of medicine and medical supplies (Fig. 9C, B7); by increasing the availability of drugs and supplies (Fig. 5C, R8), P4P reduces the likelihood of patients paying out of pocket, which increases demand. Availability of medical commodities also influences patient decision-making on membership of the community health insurance scheme, CHF (Fig. 9C, R9). When drugs and supplies are in stock, patients are more likely to register with the CHF, as they perceive services to be of better quality. Membership of the CHF also reduces the likelihood of paying out of pocket for care. The additional revenue from the CHF increases resource availability at the facility level which further increases demand for services. This cycle produces optimal behaviour when stocks of drugs and supplies are already at satisfactory levels; where they are diminished due to supply chain issues (Fig. 5B, B2) or funding (Fig. 5C, R8), this creates a vicious cycle. Lack of medicine leads to reduced payment into CHF, where community members anticipate their contributions will not guarantee availability of medicine, leading to lack of funding for medicine and supplies. An important measure to prevent this downward spiral is providers having an active, competent HFGC. In addition to advising providers on use of facility funds, the HFGC also promote community contribution into the Fund through community mobilisation and education:

"The health facility governing committee (HFGC) were not active, I remember it was in May when they were told about their roles as HFGC members. They were told why facilities run out of drugs, it just because people do not want to join CHF, I am telling you that HFGC members came up with action plan, they planed that when they go back to their villages, they are going to join CHF as well as to sensitize other village members to join CHF. This will let the community know that they must contribute for drugs."

District level stakeholder, July 2012.

## 4 Discussion

We used CLDs to provide insight into how facilities and district managers responded to P4P and shed light on mechanisms involved in provider achievement of MCH and facility reporting targets, and contextual factors supporting or impeding these. On the supply side, we observed how health worker motivation and ability of health workers to provide services were critical to achievement of P4P targets. Health worker motivation and ability to deliver services were dependant on factors directly affected by P4P (timely receipt of incentive payments, ability to purchase drugs and medical supplies using incentive payments) but importantly also on factors outside of programme influence (number of health workers, drugs and medical supplies supply chain, core facility funding). In the same vein, we observed that routine reporting of health facility data was heavily dependent on support given by the CHMT (directly influenced by P4P) but also by the composition of health workers at the facility (not directly affected by P4P). On the demand side, we observed the importance of patient perceived quality of services and community awareness of facility services (both partly influenced by P4P) in leading to a higher number of patients seeking care and facility achievement of P4P targets.

These three overarching mechanisms that resulted in provider achievement of targets are closely interconnected (Fig. 3), with changes in one part of the system leading to knock-on effects in other parts of the system. Using the CLD, it is possible to identify *catalytic variables* in the system; variables that affect multiple outcomes or mechanisms and therefore deserve careful consideration in the design of P4P schemes. Facility readiness, and especially the availability of drugs and medical supplies, is critical to service delivery. Not only in the direct sense of availability of drugs enabling health workers to deliver services but it is also critical to health worker motivation to deliver services. Facility readiness also influences patient perceived quality of facility services and feeds into the decision to seek care at the facility and decision to financially support the facility by enrolling in the community-based health insurance scheme. This variable was key to facilitating the supply and demand side mechanisms that led to facility achievement of P4P targets.

Staffing levels and supervision of facilities by district level managers are also catalytic variables. Supply of services at the facility, outreach activities (impacting demand for services) and facility reporting mechanisms (timely completion and submission of reports) only exhibited optimal behaviour where there were adequate levels of staffing at facilities. Supply of services and facility reporting mechanisms were also influenced by district management team supervision, with support leading to a more motivated workforce and facilitating provider ability to undertake routine data reporting.

The CLD also unearths potential *system levers* which are not targeted by P4P but could be incorporated to enhance the effect of the programme. CHF, the community-based health insurance scheme, was an additional source of revenue for facilities that could be used to purchase medical commodities and enhance their ability to achieve targets. We found that facilities often drew on the CHF as a lever to enhance performance. Community contributions into the CHF were dependent on community sensitisation on CHF by the HFGC, and the availability of drugs and supplies (as their absence led to out-of-pocket payments). HFGC members were not incentivised as part of P4P and yet were integral to facility success during the programme through their role in mobilising community contributions to the CHF and as signatories on facility expenditures. CHWs were also a non-incentivised group that were instrumental in stimulating demand for services at facilities, leading to facility achievement of P4P targets. CHWs provided a crucial flow of information from providers to the wider community; they were seen as trusted members of the community, able to promote health education and spread awareness on facility services and operation. The lack of incentivisation of these stakeholders sometimes undermined their leverage by facilities to achieve performance goals, where this created bad feeling. Incentivising other key stakeholders who operate at the facility and community level seems an appropriate element in the design of P4P schemes in LMICs.

This study sheds light on those P4P design features which were most important in achieving outcomes, and how programme design could be improved to enhance effects. For example, the facility-level incentive and incentivisation of district managers based on drug availability was critical to the programme resulting in the reduction in stock outs of drugs and supplies – which was a catalytic variable key to service delivery and demand for care. In settings where the availability of drugs and supplies is limited, it is essential that a share of the P4P incentive payments go to facilities to enable their procurement of drugs and supplies, and that other stakeholders that can facilitate access to drugs and supplies be incentivised as well (in this case district managers). To further strengthen the effect of the programme on drugs and supplies, and align incentives across levels of the health system, the central MSD might have been incentivised (as has been observed in the design of the most recent, scaled-up version of P4P in Tanzania) (MoHSW Ministry of Health and Social Welfare, n.d.). Delays, due to late submission of stock orders by facilities or stockouts at the MSD, may have been mitigated if the MSD had been either incentivised or supported as part of the programme design. Strengthening the supply chain of medical commodities might be assisted through other system strengthening initiatives outside of P4P, such as a redesign of logistic systems and availability of vehicles for transport of medical commodities (strategic reforms recently tested by the Global Health Fund in discussion with the Tanzania government (Githendu et al., 2020)).

Our study also identifies health system features which were critical to the supply and demand of services but were not impacted by the programme, due to their omission from the design. Staffing levels were critical to achieving outcomes, but this was largely outside of the control of facilities and districts. While district managers could reallocate staff within the district from higher to lower staffed facilities, they could not recruit new staff, even for facilities that were understaffed relative to staffing norms. Although reallocation of staff could be an incentivised target for district managers, without the capacity to hire new staff (managed at the government level and subject to restrictions on budget) this may not be a suitable target for P4P in Tanzania. Lastly, HFGC and CHW were critical stakeholders to boost demand for services, and in the case of HFGC, ensure full and effective use of facility resources, yet they were omitted from the incentive system. To maximise programme effectiveness, all stakeholders that are critical to achieving supply and demand side goals should be identified and, if possible, integrated into the incentive system. Our findings suggest that P4P as currently designed, would work best in facilities with adequate drug and supply availability and staffing levels.

Our study adds to the existing evidence base examining the effects of the P4P pilot in Tanzania on the health system and population (Mayumana et al., 2017; Anselmi et al., 2017; Binyaruka et al., 2015, 2018a; Binyaruka and Borghi, 2017; Olafsdottir et al., 2014; Binyaruka and Anselmi, 2020), by identifying those variables which are really catalytic both in terms of achieving performance targets (e.g. drugs), and limiting their achievement (staffing). The CLD also identifies pathways to improvements and potential pathways to harm (unintended negative effects), and system levers which are outside the scope of the programme but can be leveraged by providers to help achieve programme goals.

Two other studies have used CLDs to evaluate the impact of P4P programmes on health systems in low-income countries (Alonge et al., 2017; Renmans et al., 2017). A CLD of P4P in Afghanistan (Alonge et al., 2017) also identified the effect of service utilisation on facility revenue, and of health worker motivation on uptake of services. However, the Afghan CLD includes a highly a composite quality variable (representing time spent with patients, drug availability, perception of care and other measures). Our study shows these measures of quality do not necessarily move in the same direction over time and are, therefore, better observed separately. The Afghan CLD also excludes supervision and staffing which we found to be important influences on outcomes. A CLD of P4P in Uganda (Renmans et al., 2017) identified the importance of district-level supervision on health worker motivation and knowledge, as in our study, and investments in the facility environment leading to increased care seeking. However, medicines and infrastructure are combined in a ‘work environment’ variable, and unlike our study, the medicines supply chain is not included in the CLD. Reporting of health facility data was not an incentivised target in the Ugandan P4P programme and was therefore excluded from the CLD.

Singh et al. (2021) used a CLD to synthesize evidence identified in a realist review. Like our study, the realist review identifies drug availability, health worker kindness and outreach services as key mechanisms underpinning P4P effects on utilisation outcomes. Our study contributes further evidence on availability of drugs as a critical factor in community demand for community based health insurance, and the positive relationship between insurance uptake and drug availability. We also identify pathways between facility readiness and health worker motivation, and between supervision and deterrence from misreporting data.

There are a number of limitations to this study. Data used to develop the CLD were not collected for this purpose, which may have limited the degree of causal statements. However, this approach is highly cost-effective by limiting the primary data collection that is needed. As the CLD was developed by one researcher, there is a risk that unconscious bias may have gone unchecked in the CLD. However, we found the CLD to be well supported during validation. On the methods front, we could have used more objective methods to compare pairs of CLDs prior to combining them; mathematical graph theory has been used previously to compare pairs of CLDs (Markóczy, 1995; Schaffernicht and Groesser, 2011). However, because of the large number of CLDs involved, it was more practical to use qualitative reasoning methods to compare the CLDs and then combine them. While we had planned to conduct face to face validation interviews, due to COVID-19 these were conducted via Zoom. However, this online format worked effectively. To reduce the risk of recall bias stakeholders were encouraged to say when they were not confident in their recollection of events. Stakeholders often offered anecdotes and reflections to support their confirmation of model structure (or recommendation for changes) which strengthened confidence in their ability to provide evidence on their experience of the programme.

Another limitation is the generalisability of the CLD to represent pathways to impact of P4P on delivery and uptake of MCH services in other types of facilities (secondary care providers). Study authors decided to exclude secondary data collected on secondary care facilities due to the substantial programme design differences between hospitals and lower-level facilities (health centres and dispensaries), and the much larger number of primary care facilities included in the programme. Given these facility operation and design differences, the current CLD would not be generalisable to secondary care facilities.

A further limitation of this work is that we did not have data from patients themselves which may have highlighted other variables of relevance to care seeking practices. We intend to develop a system dynamics simulation model in later work that will use survey data from patients to explore the dynamic hypothesis raised in the CLD. The CLD gave an indication of variables which were more or less frequently mentioned, but it does not allow us to quantify the relative impact of different variables or loops within the system. Without quantifying relative and combined effects, it is difficult to estimate how key outcomes would be impacted by P4P design changes and understand the reasons for the dynamic behaviour playing out over time. However, in the system dynamics modelling research we have planned, we will be able to identify the key/dominant loops in the CLD by quantifying how mechanisms/loops change over time in response to P4P using the developed simulation model. The model will allow us to quantify relationships between variables and measure the effect a given loop has on key outcomes.

The CLD identified key mechanisms underpinning facility achievement of P4P targets, catalytic mechanisms impacting multiple outcomes and potential levers, and design modifications to improve programme effectiveness. Further research using CLDs to study heath systems in LMIC is urgently needed to further our understanding of how systems respond to interventions and how to strengthen systems to deliver better coverage and quality of care.

## Authors’ contributions

RC, JB, KB and NSS conceived the study. All authors contributed to the final structure and content of the paper. RC, JB, ATC, ARS, ZB and KB contributed to the determination of methods applied in this study. PB and JB managed the provision of secondary data. RC led the analysis of secondary and newly collected data in the study. RC and JM collected new data, interviews with stakeholders, to validate the causal loop diagram. RC wrote the first draft of the paper with input from JB and ZC. All authors contributed to the development of the paper and reviewed and approved the final version.

## Declaration of competing interest

None.
