# Supplementary material for: Understanding the maternal and child health system response to payment for performance in Tanzania using a causal loop diagram approach
Source: Soc Sci Med. 2021 Sep;285:114277. doi: 10.1016/j.socscimed.2021.114277 (PMC8434440; doi:10.1016/j.socscimed.2021.114277)
Supplement: Multimedia component 1 [file mmc1.docx]

# Appendix A: How to read a causal loop diagram

A CLD shows cause and effect relationships between variables in a system. The polarity indicated on each connecting arrow signifies the effect of changes in one variable on another. So, for example, if incentives result in more motivated health workers there would be a positive arrow linking ‘health worker incentive payment’ and ‘health worker motivation’ (Figure A1).

Figure A1: Example of a cause and effect relationship represented in CLD.

Delays in effect are indicated by two lines cutting through a connecting arrow (Figure A2). For example, this might apply if there were delays in payments reaching facilities.

Figure A2: Example of a delay in effect represented in CLD.


Two or more connected variables can form reinforcing (Figure A3) or balancing loops (Figure A4). For example, if incentives result in motivated health workers who then treat more patients to achieve further incentive payments, there would be positive arrows linking ‘health worker incentive payment’ to ‘health worker motivation’, ‘health worker motivation’ to ‘number of patients treated’ and ‘number of patients treated’ to ‘incentives’ forming a reinforcing loop. There exists a reinforced or amplified behaviour between these variables, a knock-on effect leading to a growing action over time. Where this loop produces desirable behaviour, it is referred to as a virtuous cycle and where undesirable behaviour persists it is referred to as a vicious cycle.

Figure A3: Example of a reinforcing loop.

In a balancing loop, the loop is prevented from exhibiting spiralling or amplified behaviour by the presence of one or more variables. For example, adequate stock of drugs enables health workers to treat patients leading to a reduction in the overall stock of drugs at the facility, is an example of a balancing loop. The loop is prevented from exhibiting amplified behaviour by one or more variables (volume of patients decreasing stock of drugs).

Figure A4: Example of a balancing loop.

Please see [1,2] for more information on how to interpret CLDs.

References

[1] Tomoaia-Cotisel A, Hyunjung K, Allen S, Blanchet K. Causal loop diagrams: a tool for visualizing emergent system behaviour. In: de Savigny D, Blanchet K, Adam T, editors. Applied systems thinking for health systems research : a methodological handbook, McGraw-Hill Education; 2017.

[2] Sterman JD. Causal loop diagrams. Business Dynamics: Systems Thinking and Modeling for a Complex World, McGraw-Hill Companies Inc; 2000.

# Appendix B: Facility and district managers indicators and performance targets during the pilot P4P programme in Tanzania

Table B1: Facility coverage, content of care and HMIS strengthening indicators and performance targets set during the pilot P4P programme in Tanzania.

| **Indicator** | **Measure** | **Baseline coverage (previous cycle)** | | | | |
| --- | --- | --- | --- | --- | --- | --- |
|  |  | **0-20%** | **21-40%** | **41-70%** | **71-85%** | **85%+** |
| *Coverage indicators* |  |  |  |  |  |  |
| % of institutional deliveries | Percentage point increase | 15% | 10% | 5% | 5% | Maintain |
| % of mothers attending a facility within 7 days of delivery | Percentage point increase | 15% | 10% | 5% | 5% | Maintain |
| % of women using long term contraceptives | Percentage point increase | 20% | 15% | 10% | Maintain above 71% | Maintain |
| % children under 1 year receiving Penta3 vaccine | Overall result | 50% | 65% | 75% | 80% +* | Maintain |
| % children under 1 year receiving measles vaccine | Overall result | 50% | 65% | 75% | 80% +* | Maintain |
| *Content of care indicators* |  |  |  |  |  |  |
| % ANC clients receiving IPT2 | Overall result | 80% | 80% | 80% | 80%+* | Maintain above 80% |
| % HIV+ ANC clients on ART | Overall result | 40% | 60% | 75% | 75%+* | Maintain |
| % of newborns receiving polio vaccine (OPV0) | Overall result | 60% | 75% | 80% | 80%+* | Maintain |
| *HMIS strengthening* |  |  |  |  |  |  |
| HMIS monthly reports correctly filled and submitted on time to CHMT | Overall result | 100% | 100% | 100% | 100% | 100% |

Notes to Table: +*80% or more. Antiretroviral therapy (ART), Antenatal care (ANC), Council Health Management Team (CHMT), Management Information System (HMIS), Human Immunodeficiency Virus (HIV), Intermittent Preventative Treatment (IPT2). Source: Binyaruka et al [1] and MoHSW [2].

Table B2: Council Health Management Team and Regional Health Management Team performance indicators set during the pilot P4P programme in Tanzania.

| **CHMT/RHMT/Both** | **Indicator** | **Measure** |
| --- | --- | --- |
|  | *Coverage indicators* |  |
| Both | % of maternal and newborn deaths that are appropriately audited on time | Overall result |
|  | *Health system strengthening* |  |
| CHMT | % of facilities reporting stock-outs of either one or more of the tracer medicines in a specified period (< 8 days) | Overall result |
|  | *HMIS strengthening* |  |
| CHMT | % of facilities included in the HMIS monthly reports exported through DHIS to RHMT in timely manner | Overall result |
|  | *Management* |  |
| RHMT | Submission to MoHSW of a Semi-Annual Regional Health Profile report, based on DHIS | Overall result |
| CHMT | % of facilities receiving a copy of a Quarterly District Health Profile report, based on DHIS | Overall result |
|  | *Overall* |  |
| Both | Overall performance along P4P facility-based indicators | Overall result |

Notes to Table: Council Health Management Team (CHMT), District Health Information Software (DHIS), Health Management Information System (HMIS), Ministry of Health and Social Welfare (MoHSW), Regional Health Management Team (RHMT). Source: MoHSW [2].

References

[1] Binyaruka P, Patouillard E, Powell-Jackson T, Greco G, Maestad O, Borghi J. Effect of Paying for Performance on Utilisation, Quality, and User Costs of Health Services in Tanzania: A Controlled Before and After Study. PLoS One 2015;10:1–16. doi:10.1371/journal.pone.0135013.

[2] MoHSW (Ministry of Health and Social Welfare), 2012. The Pwani Region Pay-for-Performance (P4P) Pilot Design Document. Dar es Salaam: MoHSW, United Republic of Tanzania.

# Appendix C: Stakeholder CLD validation interview tool

Interviewer: This series of interviews have been organised by researchers from Ifakara Health Institute and the London School of Hygiene and Tropical Medicine. We are hoping to conduct interviews with experts, such as yourself, to validate a map we have created of the Tanzania maternal and child health (MCH) system response to payment for performance (P4P). We developed the map using interview data that was collected during the pilot P4P programme in Tanzania (2011-2013); interviews were conducted with health workers, facility in-charges and district level managers on how the programme had been received by providers and managers, and what factors had facilitated or hindered effective implementation of the programme. We are currently focussing on the primary care facilities that offered MCH services and took part in the pilot programme (excluding up-graded health centres).

Interviewer: To ensure our system map accurately represents the real health system behaviour and processes that developed under the pilot we now require this map to be validated by experts with knowledge of the pilot programme.

Interviewer: During this interview, I will show you system maps that are representative of how we believe the health system functioned following the introduction of the pilot P4P programme. Using your knowledge, experience and feedback of health system operation we will then refine the structure of our maps to ensure they reflect the pilot P4P programme.

Interviewer: In the next phase of our project we are going to be looking at the differences between the pilot and other health system strengthening programmes that have taken place in the country, including the up-scaled Results-Based Financing programme (RBF, 2016-2019) and Direct Health Facility Financing programme (DHFF, 2019-Present). If you have time at the end of the interview, I would be very interested to hear your opinion on the core (intended and observed) differences in health system transformation and outcomes between the three programmes.

Interviewer: Just before we begin, I have received a copy of your consent form but I would just like to seek your verbal consent that you are happy to continue with the interview and you are happy for me to take written notes and an audio-recording of this session. This is only for our records and shared only with our research team. You can change your mind or stop the interview at any time.

Interviewer: *If no* That is okay I will take written notes instead.

*Once participant has given consent, open the Vensim diagram that shows the system map and ask the interviewee if they can see the map on their screen*

Interviewer: We have this large system map of the Tanzania health system response to P4P but to make the most use of the time we have today, I am going to focus the interview on one area of the map. The map has been split into three segments corresponding to the (i) demand, (ii) supply and (iii) reporting-side mechanisms underpinning achievement of targets during P4P, with targets represented in the diagram in bold labelled ‘Number of women and children receive incentivised services’ and ‘submission of routine health facility data by providers’.

Interviewer: In today’s interview we are going to focus on the part of the map that describes *refer to (i), (ii) or (iii)*. I have highlighted the portion of the system map that corresponds to *refer to (i), (ii) or (iii)* so that we can still see how this part of the map connects to other elements of the map (just to show it doesn’t operate in isolation). I will describe what we are seeing in the map then periodically stop to check, to your knowledge, that this process occurred during the pilot P4P programme. Your feedback will help us validate our diagram and make any necessary refinements.

Interviewer: Just a few comments on what we are seeing here. We have variables and arrows connecting each of the variables. This indicates some kind of causal relationship exists between pairs of variables. You will also notice that the arrows have polarity attached to them, plus and minus signs. These indicate the direction of causality. For example, as ‘Amount of inventive payment issued to providers’ increases, so does ‘Health worker salary top up’ (i.e. health workers receive bonus payments for improved performance during P4P).

*Stop here and check if the interviewee understands what you have described – does this make sense? *

Interviewer: You will also notice there are two small dashes across the arrow; this indicates a delay in effect. Taking the same example, although facilities who improve their performance during P4P should receive a bonus payment, there were often delays between the incentive payment being issued and health workers receiving this money (particularly at the beginning of the programme). This delay in effect is represented by those two dashes across the arrow.

*Stop here and check if the interviewee understands what you have described – does this make sense? *

*Interviewer then proceeds with taking the interviewee round the rest of this map segment, periodically stopping to check interviewee understanding and to ask if any modifications should be made to the map to reflect their experience of the programme*

*The interviewer does not have to explicitly run through these questions while discussing the map, can instead probe ‘Does this make sense? Are we missing anything important in this section of the map? Is there anything that you feel should be removed in the map?’. When an interviewee gives their feedback on the map, it will generally fall into these compartments and help the modeller to go back and make modifications to the map:*

●    Does this part of the system exist to your knowledge?

●    Are appropriate system variables represented? If not, what variables are missing or should be removed?

●    Are appropriate in and out flows represented? If not, what flows are missing or should be removed?

●    Is the polarity of in and out flows accurately represented? If not, what changes would you make?

●    Are appropriate delays in the system represented? If not, what delays are missing or should be removed?

*When interviewer has finished with validating the system map*

Interviewer: We may have already touched on this during our discussion of the map but I would also be interested to hear your view on what you think could have been changed in the implementation of the pilot programme to help facilities achieve targets (and improve the delivery and coverage of MCH services?).

Interviewer: As I said earlier, in the next phase of our project we are going to be looking at the differences between the pilot and other health system strengthening programmes that have taken place in the country, including the up-scaled Results-Based Financing programme (RBF, 2016-2019) and Direct Health Facility Financing programme (DHFF, 2019-Present). If you have time now, I would be very interested to hear your opinion on:

- What are the key similarities and differences between P4P pilot program and/or 1) RBF program and (2) DHFF program?

Interviewer: Thank you very much for your time today, this has been incredibly useful. If you feel comfortable doing so, is there anyone you would recommend for us to interview next?

# Appendix D: Mechanisms that result in changes in the supply of services, facility reporting and demand for services, highlighted in the CLD

Figure D1: Mechanisms that result in changes in the supply of services highlighted in the CLD.

Notes to Figure: Health Management Team (CHMT), Community Health Fund (CHF), Community Health Workers (CHWs), District Executive Director (DED), Health Facility Governing Committee (HGFC), Medical Stores Department (MSD), Payment for performance (P4P), Pilot Management Team (PMT).

#

Figure D2: Mechanisms that result in changes to facility reporting highlighted in the CLD.

Notes to Figure: Health Management Team (CHMT), Community Health Fund (CHF), Community Health Workers (CHWs), District Executive Director (DED), Health Facility Governing Committee (HGFC), Medical Stores Department (MSD), Payment for performance (P4P), Pilot Management Team (PMT).

Figure D3: Mechanisms that result in changes in demand for services highlighted in the CLD.

Notes to Figure: Health Management Team (CHMT), Community Health Fund (CHF), Community Health Workers (CHWs), District Executive Director (DED), Health Facility Governing Committee (HGFC), Medical Stores Department (MSD), Payment for performance (P4P), Pilot Management Team (PMT).
